# The impact of minimum wages on medical expenditures and resource misallocation: evidence from China’s healthcare system

**DOI:** 10.3389/fpubh.2025.1646631

**Published:** 2025-11-04

**Authors:** Chang Yang, Chenbing Sha, Tong An

**Affiliations:** ^1^School of Economics, Tianjin Normal University, Tianjin, China; ^2^School of Geographic and Environmental Sciences, Tianjin Normal University, Tianjin, China; ^3^School of Economics, Nankai University, Tianjin, China

**Keywords:** minimum wage, medical expenditure, hierarchical medical system, medical resource allocation, China

## Abstract

**Introduction:**

While minimum wage policies are widely advocated for promoting health equity, empirical evidence on their impact on healthcare utilization remains limited. This study provides new evidence from China, where regional minimum wages interact with a hierarchical healthcare system characterized by resource imbalances.

**Methods:**

We use large national hospital-level microdata from China to examine how regional minimum wages affect individual medical expenditures. To address endogeneity concerns, we employ an instrumental variable (IV) strategy using two-stage least squares (2SLS) estimation.

**Results:**

The IV estimates show that a one-yuan increase in the minimum wage raises outpatient spending per visit by 0.36 yuan, inpatient spending per admission by 7.90 yuan, and pharmaceutical spending per visit by 0.38 yuan. Mechanism analysis indicates three channels: higher demand for chronic disease management, greater use of treatment and surgery in inpatient care, and stronger preferences for higher-quality care, particularly in for-profit and tertiary hospitals.

**Discussion:**

We find a dual impact of minimum wage increases on the hierarchical healthcare system: higher minimum wages strengthen the gatekeeping role of primary care but simultaneously intensify inpatient demand at higher-level hospitals and in resource-concentrated regions, thereby exacerbating resource misallocation and undermining efficiency.

## 1 Introduction

Most existing studies on minimum wage policies focus on labor market outcomes such as employment rates (1), working hours (2), poverty reduction (3). While these policies aim to safeguard the living standards of low-income groups, their consequences for healthcare, an equally critical domain, remain underexplored. Recent research has begun to examine how higher wages affect health and access to care (4, 5), suggesting that income gains may ease financial barriers, expand insurance coverage (6), and in some cases reduce medical spending (7). Yet healthcare utilization has typically been treated as a secondary outcome, with little systematic attention to the behavioral mechanisms involved. This neglect arises from the indirect nature of minimum wage policies, which are not designed with healthcare in mind. It also reflects data limitations, as most studies rely on household surveys that offer only limited information on actual utilization. As a result, the causal channels linking income policy to healthcare spending and resource allocation remain insufficiently understood, particularly in developing countries where health systems are complex and resources unevenly distributed.

This paper addresses a gap in the existing literature by systematically assessing the impact of local minimum wages in China on residents’ medical expenditures, healthcare utilization, and willingness to seek care. China’s long-standing regional disparities in economic development and healthcare resources (8, 9), together with substantial cross-regional variation in minimum wage levels and enforcement, create a natural setting for this analysis. The coexistence of a hierarchical medical system and an urban–rural dual structure further provides a unique context to identify heterogeneous effects and policy transmission mechanisms.

Meanwhile, growing healthcare burdens in China have raised pressing concerns about access and affordability. Chronic disease prevalence, population aging, and shifting lifestyles have contributed to rising medical expenditures. Despite near-universal insurance^1^ coverage under the New Healthcare Reforms,^2^ financial protection remains limited: out-of-pocket payments account for 28.8% of total health spending—well above the 21% average in developed countries (10). In urban areas, 20.4% of patients requiring hospitalization went without inpatient care, with nearly half citing financial constraints (8). These challenges underscore the urgency of understanding how minimum wage policy affects treatment-seeking behavior. Given that healthcare and financial decisions are often made at the household level in China (11), wage increases may alleviate broader financial pressures and improve access to care across family members. Against this backdrop, an open question is whether income-based policies such as minimum wages can reshape healthcare utilization patterns within China’s hierarchical medical system.

In this paper, we address this question by analyzing the effects of regional minimum wages on residents’ medical expenditures using a large national microdata of hospitals and an instrumental variable (IV) strategy. The IV results show that a one-yuan increase in the minimum wage leads to a 0.36 yuan increase in outpatient expenditure per visit, a 7.90 yuan increase in inpatient expenditure per admission, and a 0.38 yuan increase in pharmaceutical expenditure per visit.

Mechanism analysis points to three primary channels. First, higher minimum wages significantly increase outpatient visits to internal medicine departments. These departments are primarily responsible for managing chronic conditions (12), suggesting that additional income helps uncover unmet demand for long-term treatment. Second, expenditures on treatment and surgery within inpatient care rise significantly and account for the largest increases among all spending categories. Third, outpatient and pharmaceutical expenditures rise more in for-profit hospitals than in nonprofit ones, indicating a preference for better service environments in routine care. Inpatient expenditures also increase more sharply in tertiary hospitals relative to primary and secondary hospitals, reflecting stronger demand for professional care in higher-tier institutions.

Our findings further reveal a dual impact of minimum wage increases on China’s hierarchical healthcare system. On the one hand, they reinforce the gatekeeping role of primary care by raising outpatient and pharmaceutical expenditures at primary and secondary hospitals, with outpatient expenditures also increasing in central and western regions where affordability constraints have been most binding. On the other hand, they intensify the concentration of inpatient demand in tertiary hospitals and in hospitals located in eastern regions where medical resources are more concentrated, thereby exacerbating resource misallocation and undermining efficiency at higher-tier institutions.

The contributions of this paper are as follows: First, it provides the first nationwide evidence from China on how minimum wage policies affect residents’ medical expenditures and healthcare utilization. We link regional minimum wage standards with large national hospital-level microdata rather than household survey data, thereby offering a more comprehensive assessment that better captures system-wide effects.

Second, the study contributes to understanding the mechanisms through which income policies shape healthcare decisions. We show that higher minimum wages increase demand for chronic disease treatment, treatment and surgery in inpatient care, and higher-quality healthcare, particularly in for-profit and tertiary hospitals, thereby expanding the prior work on the structural challenges of China’s healthcare system [e.g., (9, 13, 14)].

Third, while grounded in China, the findings contribute to a broader literature on institutional adaptation in low- and middle-income countries (LMICs). They suggest that income-based policy shocks often interact with structural constraints—such as weak primary care, fragmented service delivery, and institutional inertia, which are common across developing health systems [e.g., (15, 16)].

The rest of the paper is organized as follows. Section 2 provides an overview of the relevant institutional background in China. Section 3 outlines the data and variables used in the analysis. Section 4 presents the empirical strategy. Section 5 reports the estimation results for residents’ healthcare expenditures and explores the potential mechanisms and regional heterogeneity impact. Section 6 discusses the robustness of the results. Finally, Section 7 summarizes the findings and offers a discussion.

## 2 Institutional background

### 2.1 Minimum wage policy in China

China’s minimum wage system was institutionalized in 2004 with the promulgation of the *Minimum Wage Regulation*, which granted local governments both the authority and the responsibility to set and enforce minimum wage standards. While a prior administrative rule had existed since 1993, it lacked binding enforcement and thus had limited policy impact. The 2004 reform marked a turning point by introducing legal penalties for non-compliant employers.

Local governments generally adopt one of two approaches when determining minimum wages. The first is the Engel coefficient method, which derives a subsistence wage based on estimated food expenditure and regional Engel ratios. The second is the proportional method, which identifies low-income residents using a percentile threshold and calculates their basic living costs accordingly. A simplified variant of this proportional method was formally codified in the 2004 Minimum Wage Regulation, which stipulates that minimum wages should fall between 40 and 60% of the local average wage. This guideline provides a uniform benchmark for wage-setting across regions and has become widely adopted in practice. Both methods typically adjust for the average number of dependents per worker and incorporate additional factors such as average wages, cost of living, and required contributions to social insurance schemes.

Minimum wages in China are set at the provincial level and vary across cities within each province based on local economic development. Cities are typically grouped into three to seven tiers, each associated with a distinct wage level and subject to periodic reclassification. For example, Guangdong Province used a seven-tier system in 2004 but streamlined it to five by 2006 while simultaneously raising the wage floor across the board. To ensure that wage standards evolve with regional growth, the 2004 regulation mandated biennial adjustments. Since then, the direction of policy change has been consistently upward. In our dataset of 316 cities, 99% reported a wage increase between 2006 and 2008, confirming the widespread and systematic implementation of this adjustment mechanism.

Currently, regional minimum wage policies serve as a crucial instrument for poverty alleviation and reducing income inequality (3, 17). Higher minimum wages can enhance the social welfare of low-income populations by increasing their access to inpatient treatment and raising individual medical expenditures, thereby reducing the likelihood of foregoing medical treatment or falling into poverty due to illness.

### 2.2 Hierarchical medical system and unequal resource distribution in China

The hierarchical medical system in China is structured around a tiered diagnosis and treatment model that allocates healthcare services across three levels: primary, secondary, and tertiary institutions. Primary hospitals^3^ typically serve as the first point of contact for patients within a given community, providing basic medical care, preventive services, and rehabilitation. These facilities are embedded within local health networks and have taken on an increasingly important role in chronic disease management and population health. Secondary hospitals offer a broader range of services across larger regions, while tertiary hospitals provide highly specialized care for complex or critical cases and act as referral centers within the system.

This institutional model is designed to align patients with appropriate levels of care according to the severity of illness, thereby improving system efficiency and relieving pressure on higher-tier facilities. The effective functioning of this system hinges on primary hospitals fulfilling their gatekeeping role and absorbing routine healthcare needs. Indeed, A robust primary care infrastructure has been associated with lower healthcare costs (18) and greater system-wide efficiency.^4^

However, in practice, service utilization patterns often diverge from institutional intent. A widespread phenomenon of “inverted demand” has emerged, whereby patients bypass primary and secondary institutions and seek treatment directly at tertiary hospitals, even for minor ailments. This behavior stems in part from structural incentives embedded in the hospital classification system, which disproportionately allocates resources—such as medical equipment, pharmaceuticals, and qualified personnel—to tertiary hospitals. Over time, these facilities have concentrated the bulk of high-quality medical services, further reinforcing patient preference for higher-tier care. The result is a misalignment between service demand and institutional function, which undermines system efficiency and crowds out resources intended for more complex cases.

**Figure 1:**
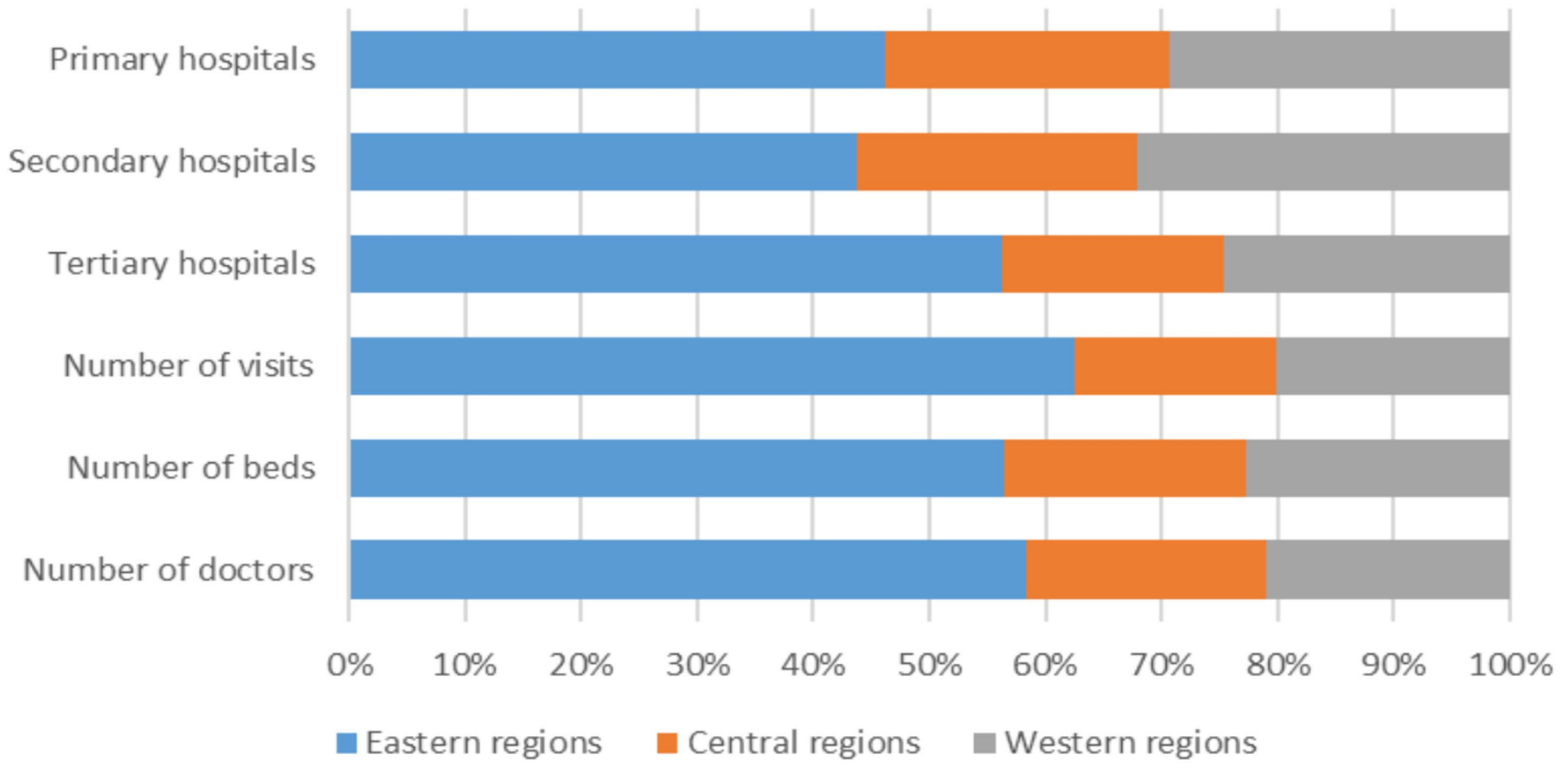
Distribution of medical resources among regions in China. This figure shows the shares of hospitals, medical personnel, and patient visits in eastern (blue), central (orange), and western (gray) regions. The eastern region accounts for about 55% of tertiary hospitals, 60% of beds and physicians, and nearly 65% of total patient visits, indicating a strong concentration of advanced medical resources, whereas central and western regions lag significantly behind in these dimensions.

Such mismatches in resource allocation are not limited to institutional tiers but are also pronounced across geographic regions. Mirroring broader patterns of uneven economic development, healthcare infrastructure is heavily concentrated in China’s more affluent eastern provinces (9, 13). As shown in Figure 1, the eastern region accounts for the largest share of tertiary hospitals, hospital beds, practicing physicians, and patient visits nationwide. In contrast, central and western regions face persistent gaps in healthcare provision, including weaker institutional capacity and underdeveloped service infrastructure. These regions often struggle to attract and retain qualified healthcare professionals, and the fragmentation of the health insurance system across provincial lines further complicates the equalization of healthcare access (10). As a result, healthcare development in the central and western regions remains significantly below the national average.

Taken together, these patterns point to a healthcare system shaped by structural imbalances—both institutional and regional. The coexistence of tier-based concentration and interregional disparities suggests a broader challenge of resource misallocation, with implications for access, equity, and efficiency. While the minimum wage is not a health-specific policy, its role in shaping household purchasing power may interact with these structural inefficiencies in nontrivial ways. By analyzing how minimum wage changes influence healthcare utilization within this uneven landscape, this study contributes to understanding the broader economic determinants of health system performance.

### 2.3 Basic medical insurance and medical burden in China

China’s Basic Medical Insurance (BMI) system was officially institutionalized in 1998 to provide coverage for urban employees and was designed as a hybrid of social pooling and individual medical savings accounts. To expand beyond the formal sector, pilot programs were launched in 2007 and scaled up in 2008 to include non-employed urban residents, such as minors and the unemployed. These pilots explored differentiated contribution mechanisms and paved the way for broader institutional integration. By 2019, over 95% of China’s population was covered by basic medical insurance, significantly enhancing social protection and improving healthcare affordability for low-income groups.^5^

Despite its broad coverage and significant improvements in access, the BMI system faces growing pressure from rising public demand and limited healthcare supply. As living standards improve and universal coverage expands, households increasingly seek higher-quality services (14). However, the supply of affordable pharmaceuticals and clinical resources has not kept pace, resulting in persistent price inflation and growing out-of-pocket expenditures. Hospitals continue to rely heavily on patient payments as a major source of revenue, weakening the protective function of insurance and making medical care less affordable for vulnerable populations.

Table 1 shows the provincial medical burden faced by low-income groups measured as the ratio of average medical expenditure to the local minimum wage. The national average stands at 27.3%, indicating that nearly a third of minimum wage income is allocated to medical care. Hubei Province bears the heaviest burden, with medical expenses accounting for 34.4% of the minimum wage, while Beijing, though the lowest among all regions, still exceeds 15%. These figures underscore the persistent affordability challenges in China’s healthcare system.

**Table 1:** Provincial medical burden in China.

Province	Medical burden	Province	Medical burden
Hubei	0.344	Liaoning	0.278
Jilin	0.339	Gansu	0.276
Henan	0.339	Sichuan	0.274
Shanxi	0.325	Guangxi	0.268
Hebei	0.317	Shaanxi	0.264
Guangdong	0.305	Qinghai	0.263
Chongqing	0.302	Inner Mongolia	0.257
Yunnan	0.299	Hainan	0.249
Heilongjiang	0.299	Tianjin	0.238
Zhejiang	0.296	Anhui	0.223
Jiangxi	0.294	Ningxia	0.222
Shandong	0.293	Xinjiang	0.216
Hunan	0.290	Shanghai	0.204
Guizhou	0.284	Tibet	0.186
Jiangsu	0.281	Beijing	0.170
Fujian	0.279	Average	0.273

## 3 Data and empirical strategy

### 3.1 Data sources

This study utilizes large national hospital-level microdata sourced from the statistical survey administered by the Ministry of Health in 2008. While the comprehensiveness of the microdata is an important advantage, its timing is equally critical. This transitional period offers a uniquely valuable setting for three reasons. First, 2008 provides a clear institutional baseline, just before the nationwide healthcare reform launched in 2009. This timing enables us to observe how wage policies interacted with the pre-reform healthcare system—prior to large-scale public investment, insurance expansion, and regulatory changes—thus allowing clearer identification of wage-policy-driven responses.

Second, the pre-reform healthcare system already exhibited structural distortions, including overreliance on tertiary hospitals, underutilization of primary care, and excessive out-of-pocket expenditures. Examining this period shows how income policy shocks interacted with these entrenched problems, providing insights into why they proved resistant to subsequent reforms.

Third, beyond these problems, many institutional features captured in 2008—such as the dominance of nonprofit hospitals and the hierarchical hospital classification—have remained remarkably stable over time. This persistence underscores the system’s institutional inertia and highlights the value of studying how socioeconomic policy shocks shaped its behavior at the onset of reform, yielding lessons on adaptability and path dependence.

The microdata covers nearly all officially registered hospitals across the country, including general hospitals, traditional Chinese medicine (TCM) hospitals, integrated TCM and Western medicine hospitals, ethnic minority hospitals, various specialized hospitals and maternal and child healthcare institutions, making the sample highly comprehensive.^6^ There are three components in the microdata. The first component provides basic hospital characteristics, including the institution’s name, address, organizational code, year of establishment, hospital tiers (tertiary, secondary, and primary) and hospital ratings (Class A, Class B, and Class C). The second component offers information on hospital resources and financial conditions, such as registered capital, number of employees, medical specialties, and the number of beds across departments. The third component reports performance and service delivery metrics, including outpatient and emergency visits, inpatient surgeries, revenues from various medical and pharmaceutical services, and over 40 indicators related to healthcare expenditures and hospital operations.

A total of 21,747 hospitals are included in this microdata. There are 1,237 tertiary hospitals, 7,235 secondary hospitals, 5,209 primary hospitals and 7,666 hospitals unrated.^7^ These hospitals are distributed across 316 cities within 31 provinces, including autonomous regions and municipalities. This coverage accounts for approximately 91.9% of all cities in China.^8^ Therefore, the sample is broadly representative of China’s hospital system.

### 3.2 Minimum wage policy

Since the implementation of the *Minimum Wage Regulation* in 2004, its effective enforcement across various regions has created an empirical setting to examine how wage policies of developing countries influence healthcare expenditures. We collected minimum wage data from 316 cities through government websites, policy documents, and official media reports. These data are then matched with the large national hospital-level microdata by city and region. To account for potential adjustment lags, we also collect regional minimum wages from 2006 and use them for robustness checks.

### 3.3 Control variables

#### 3.3.1 Hospital characteristics

We control for a set of hospital characteristics to mitigate confounding from institutional heterogeneity in medical expenditures. Specifically, the model includes hospital asset size, the number of practicing physicians, and the number of beds. We also control for hospital age, since longer-established hospitals may attract greater patient trust through reputation accumulation.

#### 3.3.2 Regional characteristics

To mitigate the impact of regional characteristics on estimation results, we control for city-level variables that may influence medical expenditures.^9^ Specifically, the model includes labor productivity, the ratio of foreign direct investment (FDI) to GDP (hereafter FDI/GDP), urban population size, the share of older adults,^10^ the education rate^11^ and ethnic minority region.^12^ Finally, we include province fixed effects to absorb unobserved institutional and socioeconomic differences across provinces that may otherwise confound the estimation.^13^

### 3.4 Outcome variables

Our primary outcome variables are defined as follows: (1) outpatient expenditure per visit, measured as hospital outpatient revenue divided by the number of outpatient visits; (2) inpatient expenditure per admission, defined as hospital inpatient revenue divided by the number of inpatient admissions; (3) pharmaceutical expenditure per visit, calculated as hospital pharmaceutical revenue divided by the number of patients receiving medication.

### 3.5 Mechanism variables

We consider the following outcome variables in our mechanism analysis: (1) Per capita visits to major hospital departments, including per capita visits to internal medicine and surgery departments. To adjust for regional population size, we use the “two-week consultation rate”^14^ as a proxy. Specifically, the number of visits to internal medicine and surgery departments in each hospital is divided by the total population in the corresponding region, and this ratio is used as the dependent variable. (2) Outpatient expenditures per visit, including registration, examination, inspection, treatment, surgical and laboratory test expenditures. Each category is calculated as the corresponding outpatient revenue divided by the total number of outpatient visits. (3) Inpatient expenditures per admission, including bed, examination, inspection, treatment, hospitalization and laboratory test expenditures. Each category is calculated as the corresponding inpatient revenue divided by the number of inpatient admissions.

### 3.6 Descriptive statistics

We matched hospital-level data with corresponding city-level variables to construct the analytical dataset. We excluded (1) hospitals with missing or abnormal values for key variables (e.g., hospitals reporting zero revenue); and (2) observations with missing or inconsistent city-level characteristic.^15^ The final analytical sample consists of 16,233 hospitals across 240 prefecture-level cities. Table 2 presents the descriptive statistics for major variables. The average outpatient expenditure per visit for residents was 87.6 yuan, while the average inpatient expenditure per admission was 2,316.2 yuan, and the average pharmaceutical expenditure per visit was 147.8 yuan.

**Table 2:** Descriptive statistics of major variables.

Category	Variables	Obs.	Mean	Std.dev.
Outcome variables	Outpatient expenditure per visit (yuan)	15,950	87.614	352.641
	Inpatient expenditure per admission (yuan)	14,946	2316.167	4081.481
	Pharmaceutical expenditure per visit (yuan)	15,897	147.772	317.371
Wage variables	Minimum wage in 2008	240	587.146	111.892
	Minimum wage in 2006	240	370.421	91.146
	Minimum wage in 2004	240	476.700	97.205
	Annual average wage of the province excluding the city itself	234	2015.794	281.269
Mechanism variables	Per capita visits to internal medicine (per 10,000 people)	13,155	73.637	156.969
	Per capita visits to surgery department (per 10,000 people)	12,538	30.681	66.035
	Average outpatient registration expenditure per visit (yuan)	13,465	1.751	5.692
	Average outpatient examination expenditure per visit (yuan)	12,438	4.331	24.941
	Average outpatient inspection expenditure per visit (yuan)	14,930	22.158	43.968
	Average outpatient laboratory test expenditure per visit (yuan)	14,488	10.986	35.793
	Average outpatient treatment expenditure per visit (yuan)	15,098	25.848	97.279
	Average outpatient surgery expenditure per visit (yuan)	12,872	11.713	88.784
	Average inpatient bed expenditure per admission (yuan)	14,035	351.234	1335.715
	Average inpatient examination expenditure per admission (yuan)	11,917	95.349	414.556
	Average inpatient examination inspection per admission (yuan)	13,870	232.795	373.599
	Average inpatient examination laboratory test per admission (yuan)	13,587	232.152	339.074
	Average inpatient examination treatment per admission (yuan)	14,209	724.136	1474.244
	Average inpatient examination surgery per admission (yuan)	11,006	1507.183	2312.732
Hospital characteristics	Hospital asset scale (10 thousand yuan)	16,233	1998.532	15552.830
	Number of practicing physicians	16,233	54.741	88.581
	Number of beds	16,233	155.419	222.812
	Hospital age (year)	16,233	28.866	21.944
City characteristics	Labor productivity (10 thousand yuan)	240	14.786	6.243
	FDI/GDP	240	0.028	0.077
	Population size (10 thousand)	240	432.296	308.878
	Share of older adults	240	0.130	0.599
	Education rate	240	0.951	0.029
	Ethnic minority region	240	0.092	0.289

The units for minimum wage, average wage, and various types of medical expenditure per resident are in RMB (yuan); the units for per capita visits to internal medicine and surgery departments are visits per 10 thousand people; the units for hospital asset size and labor productivity in cities are in 10 thousand yuan; and the unit for city population size is in 10 thousand people. Statistical results are presented with three decimal places.

**Figure 2:**
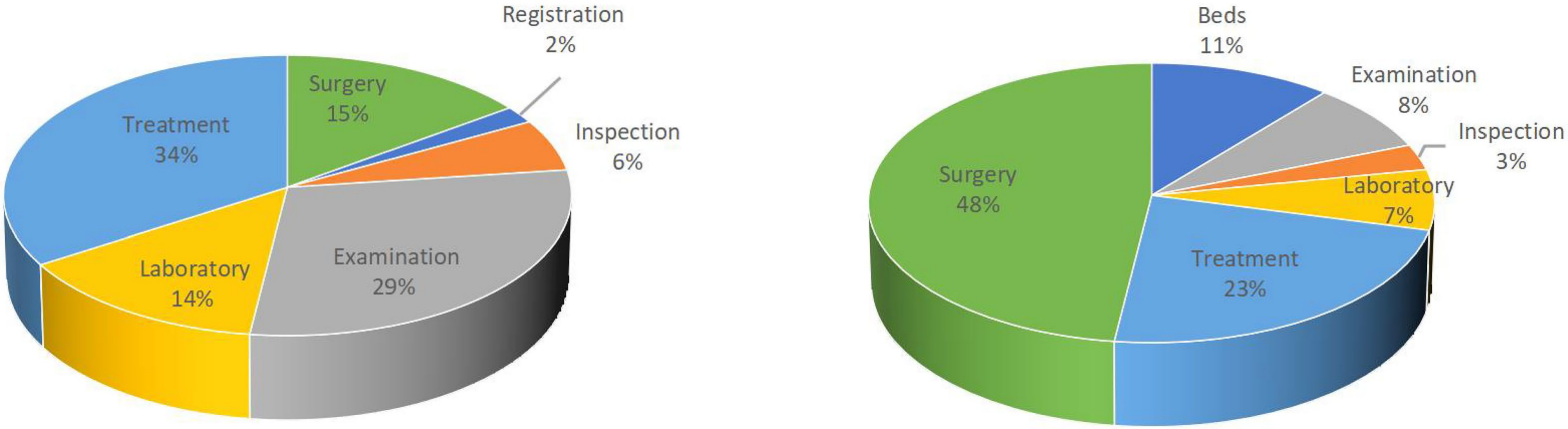
Breakdown of outpatient and inpatient expenditure per visit in China. The left figure shows outpatient expenditure, dominated by treatment (34%) and examinations (29%), with surgery and laboratory services accounting for smaller shares. The right figure shows inpatient expenditure, where surgery makes up nearly half of total costs (48%), followed by treatment (23%) and bed charges (11%). Percentages indicate the share of each item in total outpatient or inpatient expenditure.

Figure 2 presents the expenditure shares of various categories for outpatient and inpatient care. The left figure shows that treatment and examination dominate outpatient expenditures, accounting for 34% and 29%, respectively, together exceeding 60% of total costs. In contrast, the right figure shows that surgery constitutes the largest share of inpatient expenditures, with an average per-admission cost of 1,490 yuan, representing 48% of total inpatient spending. To put these figures into perspective, the national minimum wage in 2008 averaged 587.4 yuan per month (7,048.8 yuan annually). Thus, the average cost of a single inpatient surgery accounted for 21% of an individual’s annual minimum wage income, underscoring the substantial financial burden healthcare imposes on low-income households.

Table 3 provides a decomposition of per capita patient expenditures across hospitals by tier (Panel A) and ownership type (Panel B). In Panel A, average expenditures in primary and secondary hospitals are broadly comparable, particularly for inpatient and pharmaceutical services, whereas spending in tertiary hospitals is substantially higher. Notably, inpatient and pharmaceutical expenditures in tertiary hospitals are nearly double those in secondary hospitals. This pattern reflects the excessive demand for higher-tier hospitals for relatively minor conditions within China’s hierarchical medical system, where the gatekeeping role of primary care remains weak. Such misallocation contributes to inefficiencies in service delivery and resource waste.

**Table 3:** Outcomes of hospitals with heterogeneity.

Variables	Obs.	Mean	Std.dev.
Panel A: Hospitals by tier			
Outpatient expenditure per visit (yuan)			
Primary hospitals	9,107	95.381	439.470
Secondary hospitals	5,817	73.196	193.814
Tertiary hospitals	1,026	100.422	64.589
Inpatients expenditure per admission (yuan)			
Primary hospitals	8,126	2025.393	4230.829
Secondary hospitals	5,794	2297.365	3864.820
Tertiary hospitals	1,026	4725.301	3177.867
Pharmaceutical expenditure per visit (yuan)			
Primary hospitals	9,062	125.351	350.206
Secondary hospitals	5,810	155.542	216.583
Tertiary hospitals	1,025	301.947	428.565
Panel B: Hospitals by ownership type			
Outpatient expenditure per visit (yuan)			
Non-profit hospitals	12,811	68.399	265.811
For-profit hospitals	3,139	166.039	579.614
Inpatients expenditure per admission (yuan)			
Non-profit hospitals	12,195	2284.239	3915.936
For-profit hospitals	2,751	2457.702	4744.529
Pharmaceutical expenditure per visit (yuan)			
Non-profit hospitals	12,765	145.321	268.073
For-profit hospitals	3,132	157.761	467.214

Panel B shows that for-profit hospitals account for roughly one-fifth of all hospitals in the dataset. Across all categories—outpatient, inpatient, and pharmaceutical—average expenditures are consistently higher in for-profit hospitals compared to their nonprofit counterparts. This pattern is consistent with institutional arrangements in China’s healthcare system, where public non-profit hospitals operate under government-administered fee schedules and fiscal subsidies, while for-profit hospitals retain greater autonomy to set market-based prices and are often excluded from public insurance reimbursement.

## 4 Empirical strategy

### 4.1 Empirical methods: IV/2SLS

This paper aims to estimate the causal effect of regional minimum wage levels on medical expenditure of patients. We start with a linear model as shown in Equation 1.


(1) $$E_{ijk}=\beta_{0}+\beta_{1}Mwage_{jk}+\beta_{2}Hospital_{ijk}+\beta_{3}City_{jk}+\phi_{k}+ui_{jk}$$

$E_{ijk}$ denotes the per capita expenditure of patients in city j, province k. $Mwage_{jk}$ is the regional minimum wage. $\beta_{1}$ captures the effect of minimum wage on medical expenditure, which is the main parameter of interest. $Hospital_{ijk}$ is a vector of hospital-level characteristics, including hospital asset scale, number of practicing physicians and beds, as well as hospital age. And $City_{jk}$ is a vector of city-level characteristic, including labor productivity, scale of FDI and population size. $\phi_{k}$ represents the fixed effects, accounting for heterogeneity across provinces. We specify the error term to have a hospital and regional effect as well as a white noise error $u_{ijk}$ .

### 4.2 Endogeneity issues

Our estimates face potential endogeneity concerns, primarily arising from two sources. First, reverse causality between local minimum wages and medical expenditures may bias the estimates. In economically developed cities, residents are more likely to seek formal treatment for chronic conditions due to better healthcare systems. This can improve household health, reduce absenteeism, and ultimately raise regional income levels (19). In addition, optimistic expectations about macroeconomic conditions may encourage more proactive healthcare utilization, further boosting labor productivity and stimulating economic growth. Since minimum wages are typically set in relation to economic conditions, regional development levels play a key role in shaping local minimum wage policies. Second, omitted variable bias may arise if unobserved city-level factors —such as local development strategies, demographic trends, or healthcare infrastructure—jointly affect both minimum wage policies and healthcare utilization.

The determination of local minimum wage standards in China is largely shaped by structural institutional factors, including historical labor market conditions, local administrative capacity, and province-level policy coordination. These factors influence wage-setting processes and constitute the core drivers of minimum wage levels. Crucially, after controlling for contemporaneous socioeconomic indicators (e.g., GDP, income levels) and healthcare-related characteristics (e.g., infrastructure, insurance coverage), these institutional determinants are unlikely to directly affect current medical expenditures through other channels.

Based on this logic, we construct two IVs. The first IV (*IV_1_*) is the regional minimum wage level in 2004—the first year of nationwide enforcement of the *Minimum Wage Regulation*^16^—following Mayneris et al. (20). *IV_1_* reflects historical wage-setting practices shaped by early labor market structures and administrative capacity, and is strongly correlated with subsequent minimum wage adjustments. Its temporal separation from the outcome period also makes it unlikely to be influenced by contemporaneous healthcare behaviors or expenditures, thereby supporting both the relevance and exogeneity conditions required for a valid instrument.

According to the *Minimum Wage Regulation*, the statutory minimum wage should be set between 40 and 60% of the regional average wage. Following Mayneris et al. (20) and Zhang et al. (21), we construct the second IV (*IV_2_*) as 40% of the average wage in other cities within the same province, excluding the city itself. *IV_2_* is defined as follows:


(2) $$IV_{j}=\frac{\overset{n}{\underset{i\neq j}{\sum }}Average\_wage_{i}}{n-1}\times 40\%$$

i,j denote two different cities, n denotes the number of cities in a same province. $Average\_wage_{i}$ is the average wage of city i . Equation 2 indicates that *IV_2_* of city j is determined by other cities in the same province.^17^

This cross-city average serves as a strong predictor of local minimum wages, satisfying the relevance condition. At the same time, the instrument excludes the city itself and reflects wage levels shaped by broader provincial labor dynamics. As a result, it is unlikely to be directly correlated with city-specific medical expenditures, which are mainly determined by local income levels, demographic profiles, and healthcare service availability. These features strengthen the plausibility of the exclusion restriction.

Since using multiple IVs in large samples improves estimation efficiency (22), we employ these two IVs in the 2SLS estimation. We further assess their validity by conductiong validity by conducting a weak identification test (Kleibergen-Paap F-statistics), an underidentification test (LM statistics), and an overidentification test (Hansen J statistics). Results from all IV regressions are reported in the main tables, with robust standard errors throughout.

## 5 Estimation results

### 5.1 Main results on residents’ medical expenditure

Table 4 presents the main results with province fixed effects. Column (1) reports the OLS estimates from Equation (1), controlling for a range of hospital and city characteristics as well as province fixed effects. The estimate show a statistically significant association between the minimum wage and outpatient expenditure per visit. To address potential endogeneity, Column (2) reports the IV-2SLS estimates. The coefficient increases in magnitude and remains statistically significant at the 1% level, indicating that OLS may underestimate the effect.

**Table 4:** Main results on residents’ healthcare expenditures.

Variables	(1)	(2)	(3)	(4)	(5)	(6)
	OLS	IV	OLS	IV	OLS	IV
	Outpatient expenditure per visit		Inpatient expenditure per admission		Pharmaceutical expenditure per visit	
Minimum wage	0.2623***	0.3553***	6.0784***	7.8952***	0.2627***	0.3752***
	(0.0405)	(0.0595)	(0.3834)	(0.4946)	(0.0365)	(0.0484)
Observations	15,950	14,505	14,946	13,661	15,897	14,452
Hospital-level controls	Yes	Yes	Yes	Yes	Yes	Yes
City-level controls	Yes	Yes	Yes	Yes	Yes	Yes
Province FE	Yes	Yes	Yes	Yes	Yes	Yes
First stage F-stats		10454.479		9784.3537		10406.183
*p* value of LM statistic		0.0000		0.0000		0.0000
*p* value of Hansen J statistic		0.2320		0.4559		0.8198

***p < 0.01, **p < 0.05, *p < 0.1. Numbers in parentheses are robust standard errors. Hospitals located in municipalities directly under the central government and Tibet Autonomous Region are excluded from the IV regressions. The Stock-Yogo weak identification test critical value for 10% maximal IV size is 19.93.

Column (3) shows that the effect of the regional minimum wage on inpatient expenditure per admission is substantially larger than its effect on outpatient expenditure per visit. This result is robust to the IV-2SLS estimation and remains significant at the 1% level (see Column 4). Columns (5) and (6) further demonstrate that higher minimum wages significantly increase pharmaceutical expenditure per visit, regardless of the estimation method.

Based on the 2SLS results, a one-yuan increase in the minimum wage is associated with a 0.36 yuan increase in outpatient expenditure per visit, an 7.90 yuan increase in inpatient expenditure per admission, and a 0.38 yuan increase in pharmaceutical expenditure per visit. According to statistics from the MOH, in 2008 the average resident had 2.7 outpatient visits and 0.087 inpatient admissions per year.^18^ Using these figures, a 1 yuan increase in the minimum wage corresponds to an annual increase of approximate 0.96 yuan in outpatient expenditure per capita, 0.67 yuan in inpatient expenditure per capita, and 1.01 yuan in pharmaceutical expenditure per capita.

For statistical inference, we report test statistics and corresponding *p*-values for weak identification and under-identification. The weak identification test is based on the Kleibergen-Paap rk statistic, and the under-identification test relies on the LM statistic. In all IV regressions involving outpatient, inpatient, and pharmaceutical expenditures, the Kleibergen-Paap rk F-statistics exceed 19.93, well above the Stock-Yogo critical value for a 10% maximal IV size. In addition, the under-identification null is rejected, confirming the relevance of the two instruments (23, 24). The Hansen J-statistics from the over-identification tests futher support the validity of the exclusion restrictions.

### 5.2 Mechanisms

#### 5.2.1 Demand for chronic disease treatment

A key mechanism behind the observed effects is healthcare utilization for chronic diseases. In contrast to infectious diseases, acute conditions, or accidents—which often require immediate medical attention regardless of income—chronic illnesses typically involve long duration, high prevalence, substantial costs, and elevated risks of disability or mortality (25). It is therefore plausible that minimum wage policies have their strongest influence on spending related to chronic conditions, which demand sustained treatment and long-term care.

By raising overall wage levels within a region (19), a higher minimum wage enhances households’ financial capacity to cover medical expenses, including specialized procedures and prescription drugs. This increased capacity enables low-income individuals to seek treatment in formal medical institutions rather than delaying or forgoing care, thereby improving the continuity of chronic disease management.

Moreover, given that healthcare decisions in China are often made at the household level and medical expenses are typically shared among family members (11), higher minimum wages can ease the financial burden on other household members. This intra-household reallocation of resources may further suppot expenditures on higher-quality healthcare services and medications, reinforcing demand for long-term treatment.

To empirically test this mechanism, we rely on patterns of utilization across medical departments. Although the dataset does not contain disease-specific expenditure information, the categorization of patient visits by clinical department provides a useful proxy. In China, hospitals are broadlly categorized into internal medicine and surgery. Internal medicine primarily treats chronic illnesses requiring prolonged care and symptom management, such as cerebrovascular diseases, cancer, respiratory conditions, and cardiovascular diseases—all leading causes of death in China (12, 26). By contrast, surgical departments mainly treat acute conditions that are curable in the short term through operative procedures, including orthopedic cases (e.g., fractures) and neurosurgical emergencies (e.g., traumatic brain injuries). While detailed revenue data by department are unavailable, the dataset includes the number of patient visits by department, providing a feasible strategy to examine whether minimum wage policies influence healthcare utilization for chronic conditions.

Table 5 reports the estimation results for per capita visits to internal medicine and surgical departments among urban residents. The OLS estimates in Columns (1) and (3) show that increases in the minimum wage are significantly and positively associated with visit rates at the 1% level. The estimated effect is stronger for internal medicine than for surgical department, consistent with a greater sensitivity of visits related to chronic and long-term conditions. After addressing potential endogeneity, the IV-2SLS estimates in Columns (2) and (4) remain positive and statistically significant for internal medicine visits, while the effect for surgical visits declines in magnitude and is significant only at the 5% level. These findings suggest that higher minimum wages primarily stimulate healthcare utilization for chronic disease management, while their effect on sugical demand is relatively limited. Moreover, the vary large first-stage F-statistics and the Hansen J test indicate that the IV is both highly relevant and not rejected by overidentification tests.

**Table 5:** Effects on per capita visits to major hospital departments.

Variables	(1)	(2)	(3)	(4)
	OLS	IV	OLS	IV
	Per capita visits to internal medicine		Per capita visits to surgical department	
Minimum wage	0.0669***	0.1036***	0.0344***	0.0207**
	(0.0166)	(0.0222)	(0.0089)	(0.0096)
Observations	13,155	12,011	12,538	11,474
Hospital-level controls	Yes	Yes	Yes	Yes
City-level controls	Yes	Yes	Yes	Yes
Province FE	Yes	Yes	Yes	Yes
First stage F-stats		8690.077		8277.0255
*p* value of LM statistic		0.0000		0.0000
*p* value of Hansen J statistic		0.2004		0.4014

***p < 0.01, **p < 0.05, *p < 0.1. Numbers in parentheses are robust standard errors. Hospitals located in municipalities directly under the central government and Tibet Autonomous Region are excluded from the IV regressions. The Stock-Yogo weak identification test critical value for 10% maximal IV size is 19.93.

#### 5.2.2 Demand for treatment and surgery in inpatient care

The second mechanism concerns the induced expenditures associated with costly treatment and surgery in inpatient care. In 2009, the average out-of-pocket inpatient expenses per admission for common chronic diseases in China accounted for 50% of the annual disposable income of urban residents (approximately USD 750 per capita) and 1.3 times the annual income of rural residents (approximately USD 291 per capita) (27). Among all costs, inpatient treatment and surgery represent the most expensive components. Individuals with low socio-economic status often find it difficult to afford costly inpatient care, leading them to forgo necessary hospitalizations and surgeries. A higher minimum wage relaxes affordability constraints on inpatient treatment and surgery, thereby enabling utilization of services that would otherwise be postponed or forgone—particularly those related to chronic disease management and non-emergency procedures.

Table 6 presents the IV-2SLS estimation results for inpatient expenditures by categories. The effects are positive and statistically significant across all items, but the largest coefficients are concentrated in treatment and surgery (Columns 5 and 6), where a one-yuan increase in the minimum wage raises spending by 2.97 yuan and 3.06 yuan, respectively. This confirms that wage gains translate most strongly into utilization of the costliest inpatient services.

**Table 6:** Effects on inpatient expenditure per admission by category.

Variables	(1)	(2)	(3)	(4)	(5)	(6)
	Beds expenditure	Examination expenditure	Inspection expenditure	Laboratory test expenditure	Treatment expenditure	Surgery expenditure
Minimum wage	1.5266***	0.2271***	0.6376***	0.6704***	2.9682***	3.0638***
	(0.1796)	(0.0421)	(0.0521)	(0.0491)	(0.2247)	(0.3584)
Observations	12,816	10,799	12,677	12,415	12,988	10,117
Province FE	Yes	Yes	Yes	Yes	Yes	Yes
Hospital Controls	Yes	Yes	Yes	Yes	Yes	Yes
City controls	Yes	Yes	Yes	Yes	Yes	Yes
First stage F-stat	9080.4042	7406.7607	9009.8617	8749.0604	9285.4221	6936.2522
*p* value of LM statistics	0.0000	0.0000	0.0000	0.0000	0.0000	0.0000
*p* value of Hansen J statistic	0.9987	0.9583	0.3453	0.7544	0.2069	0.7027

Only IV estimation results are presented. ***p < 0.01, **p < 0.05, *p < 0.1. The dependent variable measures inpatient expenditure per admission by category. Numbers in parentheses are robust standard errors. Hospitals located in municipalities directly under the central government and Tibet Autonomous Region are excluded from the IV regressions. The Stock-Yogo weak identification test critical value for 10% maximal IV size is 19.93.

Table 7 presents the IV-2SLS estimates for outpatient expenditures. Treatment and surgery show the largest responses again, yet the magnitudes remain much smaller than those observed for inpatient care. The results indicate that minimum wage policies have their strongest impact on inpatient treatment and surgery, as wage increases relax affordability constraints in these high-cost services and thereby release previously suppressed demand—particularly for chronic non-communicable diseases.^19^

**Table 7:** Effects on outpatient expenditure per visit by category.

Variables	(1)	(2)	(3)	(4)	(5)	(6)
	Registration expenditure	Examination expenditure	Inspection expenditure	Laboratory test expenditure	Treatment expenditure	Surgery expenditure
Minimum wage	0.0032***	0.0106***	0.0096	0.0241***	0.1493***	0.0757***
	(0.0009)	(0.0027)	(0.0068)	(0.0059)	(0.0164)	(0.0164)
Observations	12,193	11,250	13,586	13,167	13,721	11,699
Province FE	Yes	Yes	Yes	Yes	Yes	Yes
Hospital Controls	Yes	Yes	Yes	Yes	Yes	Yes
City controls	Yes	Yes	Yes	Yes	Yes	Yes
First stage F-stat	8628.8865	7848.9191	9798.6654	9399.1914	9866.9869	8296.5514
p value of LM statistic	0.0000	0.0000	0.0000	0.0000	0.0000	0.0000
p value of Hansen J statistic	0.4929	0.275	0.7073	0.1984	0.913	0.8298

Only IV estimation results are presented. ***p < 0.01, **p < 0.05, *p < 0.1. The dependent variable measures outpatient expenditure per visit by category. Numbers in parentheses are robust standard errors. Hospitals located in municipalities directly under the central government and Tibet Autonomous Region are excluded from the IV regressions. The Stock-Yogo weak identification test critical value for 10% maximal IV size is 19.93.

#### 5.2.3 Demand for higher-quality healthcare

The third mechanism relates to residents’ demand of higher-quality healthcare, which can take the form of either better treatment environments or access to more advanced medical resources. We examine this channel along two institutional dimensions: hospital ownership and hospital tier.

##### 5.2.3.1 Choice of for-profit hospitals

Hospitals in China are classified into two types based on ownership: non-profit and for-profit. Non-profit hospitals typically receive government subsidies and tax exemptions, which help keep their service prices low and ensure broad coverage under public health insurance. In contrast, for-profit hospitals are fewer in number,^20^ and the actual prices for pharmaceuticals and medical services are generally much higher than those at non-profit hospitals. Moreover, most for-profit hospitals are not included in the public health insurance network, meaning that patients often face high out-of-pocket costs—or must cover the full amount themselves.

Beyond price differentials, variation in service quality also shapes patient choices. Policy guidance has emphasized that private hospitals should position themselves through differentiated and higher-end services, a direction reflected in patient perceptions. For instance, a satisfaction survey in Shenzhen in 2024 found that dissatisfaction with waiting times was markedly higher in public hospitals than in private ones. Supporting evidence from Eggleston et al. (28) further shows that for-profit hospitals in China often differentiate themselves through appointment-based consultations and modernized facilities, thereby offering a more convenient and patient-centered care environment.

Table 8 reports the heterogeneous effects by hospital ownership, with Panel A presenting results for for-profit hospitals and Panel B for non-profit hospitals. The IV-2SLS estimates show that minimum wage increases significantly raise inpatient expenditures in both types of hospitals, suggesting that wage gains generally expand demand for costly hospitalizations regardless of ownership. For outpatient and pharmaceutical spending, however, the effects are much larger in for-profit hospitals. This indicates that higher minimum wages lead patients to shift a greater share of their outpatient consultations and medication purchases toward for-profit providers. Such behavior reflects a pursuit of better service environments—shorter waiting times and more personalized care—rather than purely lower prices.

**Table 8:** Identifying the heterogeneity mechanism by hospital ownership.

Variables	(1)	(2)	(3)	(4)	(5)	(6)
	OLS	IV	OLS	IV	OLS	IV
	Outpatient expenditure per visit		Inpatient expenditure per admission		Pharmaceutical expenditure per visit	
Panel A: For-profit hospitals						
Minimum wage	0.6598***	0.7419***	5.9551***	7.5741***	0.4959***	0.6849***
	(0.1404)	(0.2157)	(1.0141)	(1.3728)	(0.1379)	(0.1863)
Observations	3,139	2,738	2,751	2,433	3,132	2,730
Hospital-level controls	Yes	Yes	Yes	Yes	Yes	Yes
City-level controls	Yes	Yes	Yes	Yes	Yes	Yes
Province FE	Yes	Yes	Yes	Yes	Yes	Yes
First stage F-stat		1150.8821		819.688		1151.4825
*p* value of LM statistic		0.0000		0.0000		0.0000
*p* value of Hansen J statistic		0.8982		0.8479		0.2833
Panel B: Non-profit hospitals						
Minimum wage	0.1703***	0.2515***	6.2141***	7.9057***	0.2316***	0.3213***
	(0.0411)	(0.0628)	(0.4166)	(0.5535)	(0.0336)	(0.0437)
Observations	12,811	11,767	12,195	11,228	12,765	11,722
Hospital-level controls	Yes	Yes	Yes	Yes	Yes	Yes
City-level controls	Yes	Yes	Yes	Yes	Yes	Yes
Province FE	Yes	Yes	Yes	Yes	Yes	Yes
First stage F-stats		8846.5923		8294.5588		8795.0898
*p* value of LM statistic		0.0000		0.0000		0.0000
*p* value of Hansen J statistic		0.2065		0.5754		0.2027

***p < 0.01, **p < 0.05, *p < 0.1. Numbers in parentheses are robust standard errors. Hospitals located in municipalities directly under the central government and Tibet Autonomous Region are excluded from the IV regressions. The Stock-Yogo weak identification test critical value for 10% maximal IV size is 19.93.

##### 5.2.3.2 Preference for higher-tier hospitals

Another possible mechanism stems from China’s hierarchical medical system. As previously discussed, this system is intended to enhance overall service efficiency by distributing healthcare resources across different tiers of institutions. In practice, however, high-quality medical resources are heavily concentrated in tertiary hospitals, which leads many patients to bypass lower-tier facilities in favor of tertiary care—resulting in inefficient resource utilization (29). When the minimum wage rises, individuals’ ability to afford out-of-pocket healthcare expenses improves.

Table 9 provides separate estimates by hospital tier and reveals distinct patterns across service types. For outpatient expenditures and pharmaceutical expenditures, the effect of the minimum wage is strongest in primary hospitals and weakens with higher tiers, becoming small but insignificant in tertiary hospitals. This gradient indicates that wage increases primarily alleviate the financial barriers underlying the phenomenon of “forgoing care for minor illnesses”, thereby releasing suppressed demand for basic outpatient services and channeling patients more effectively toward primary facilities.

**Table 9:** Identifying the heterogeneity mechanism by hospital tier.

Variables	(1)	(2)	(3)	(4)	(5)	(6)
	OLS	IV	OLS	IV	OLS	IV
	Outpatient expenditure per admission		Inpatient expenditure per admission		Pharmaceutical expenditure per visit	
Panel A: Primary hospitals						
Minimum wage	0.3681***	0.5125***	5.2761***	7.1373***	0.2973***	0.4359***
	(0.0774)	(0.1085)	(0.6524)	(0.8270)	(0.0600)	(0.0799)
Observations	9,107	8,244	8,126	7,423	9,062	8,197
Hospital-level controls	Yes	Yes	Yes	Yes	Yes	Yes
City-level controls	Yes	Yes	Yes	Yes	Yes	Yes
Province FE	Yes	Yes	Yes	Yes	Yes	Yes
First stage F-stats		5494.4733		4962.0047		5463.5215
p value of LM statistic		0.0000		0.0000		0.0000
p value of Hansen J statistic		0.1380		0.7791		0.2283
Panel B: Secondary hospitals						
Minimum wage	0.1464***	0.1597***	6.8606***	8.3763***	0.2342***	0.3258***
	(0.0349)	(0.0327)	(0.4532)	(0.6135)	(0.0307)	(0.0429)
Observations	5,817	5,376	5,794	5,354	5,810	5,370
Hospital-level controls	Yes	Yes	Yes	Yes	Yes	Yes
City-level controls	Yes	Yes	Yes	Yes	Yes	Yes
Province FE	Yes	Yes	Yes	Yes	Yes	Yes
First stage F-stats		3849.7555		3784.5946		3843.8733
*p* value of LM statistic		0.0000		0.0000		0.0000
*p* value of Hansen J statistic		0.1680		0.2959		0.3611
Panel C: Tertiary hospitals						
Minimum wage	0.0370	0.0927**	7.3601***	8.9333***	0.4436*	0.4806
	(0.0430)	(0.0443)	(1.1094)	(1.1874)	(0.2417)	(0.3193)
Observations	1,026	885	1,026	884	1,025	885
Hospital-level controls	Yes	Yes	Yes	Yes	Yes	Yes
City-level controls	Yes	Yes	Yes	Yes	Yes	Yes
Province FE	Yes	Yes	Yes	Yes	Yes	Yes
First stage F-stats		305.5263		304.7456		305.5263
*p* value of LM statistic		0.0000		0.0000		0.0000
*p* value of Hansen J statistic		0.7949		0.055		0.1557

***p < 0.01, **p < 0.05, *p < 0.1. Numbers in parentheses are robust standard errors. Hospitals located in municipalities directly under the central government and Tibet Autonomous Region are excluded from the IV regressions. The Stock-Yogo weak identification test critical value for 10% maximal IV size is 19.93.

By contrast, the coefficients for inpatient treatment rise with hospital tier, with the largest effect in tertiary hospitals (8.93 yuan per admission), far exceeding those in primary and secondary hospitals. This indicates that for complex or costly services, patients continue to prefer tertiary hospitals once affordability constraints are relaxed.

Taken together, the findings point to a dual impact of minimum wage policies on China’s hierarchical healthcare system. On the one hand, they stimulate outpatient utilization at lower-level facilities, partially reinforcing the system’s intended role of channeling common illnesses to the grassroots. On the other hand, they disproportionately increase demand for high-cost inpatient services in tertiary hospitals, aggravating the “demand inversion” whereby tertiary hospitals absorb cases that could otherwise be treated at lower tiers.

### 5.3 Heterogeneity impact among regions

China’s healthcare resources are unevenly distributed, with eastern regions generally enjoying greater availability than central and western regions (9, 30). To address potential biases stemming from these regional disparities, Table 10 presents the estimates for eastern (Panel A) and central and western (Panel B) regions. While the direction of the effects is consistent across regions, their magnitudes differ: outpatient expenditures respond more strongly in the central and western regions, whereas inpatient and pharmaceutical expenditures show larger effects in the eastern regions.

**Table 10:** Robustness to heterogeneity impact among regions in China.

Variables	(1)	(2)	(3)	(4)	(5)	(6)
	OLS	IV	OLS	IV	OLS	IV
	Outpatient expenditure per visit		Inpatient expenditure per admission		Pharmaceutical expenditure per visit	
Panel A: Eastern regions						
Minimum wage	0.2020***	0.3606***	6.4931***	9.2008***	0.3215***	0.4553***
	(0.0448)	(0.0851)	(0.4944)	(0.7090)	(0.0518)	(0.0745)
Observations	7,788	6,944	7,166	6,464	7,766	6,919
Hospital-level controls	Yes	Yes	Yes	Yes	Yes	Yes
City-level controls	Yes	Yes	Yes	Yes	Yes	Yes
Province FE	Yes	Yes	Yes	Yes	Yes	Yes
First stage F-stats		4131.1639		3815.0086		4109.8608
p value of LM statistic		0.0000		0.0000		0.0000
p value of Hansen J statistic		0.9637		0.4738		0.672
Panel B: Central and western regions						
Minimum wage	0.3533***	0.4039***	6.3570***	7.8093***	0.2917***	0.4016***
	(0.0678)	(0.0900)	(0.5699)	(0.7323)	(0.0529)	(0.0626)
Observations	8,162	7,561	7,780	7,197	8,131	7,533
Hospital-level controls	Yes	Yes	Yes	Yes	Yes	Yes
City-level controls	Yes	Yes	Yes	Yes	Yes	Yes
Province FE	Yes	Yes	Yes	Yes	Yes	Yes
First stage F-stats		13218.474		12356.779		13122.908
p value of LM statistic		0.0000		0.0000		0.0000
p value of Hansen J statistic		0.0941		0.7561		0.5697

***p < 0.01, **p < 0.05, *p < 0.1. Numbers in parentheses are robust standard errors. Hospitals located in municipalities directly under the central government and Tibet Autonomous Region are excluded from the IV regressions. The Stock-Yogo weak identification test critical value for 10% maximal IV size is 19.93.

This contrast reflects differences in both healthcare infrastructure and economic development. In the central and western regions, where medical facilities are less developed and incomes lower, wage increases primarily ease the burden of basic outpatient care, activating latent demand for minor illnesses that was previously forgone due to cost. By contrast, in the more affluent eastern regions, with better-equipped hospitals and broader insurance coverage, the effects are concentrated in inpatient and pharmaceutical spending, suggesting that higher incomes are more readily converted into the use of high-cost services.

These regional patterns also reveal implications for China’s hierarchical medical system. In the central and western regions, the wage-induced increase in outpatient spending—especially at lower-tier facilities—suggests a reinforcement of the system’s intended function of managing common illnesses at the primary-care level. In contrast, the stronger growth of inpatient and pharmaceutical expenditures in the east, particularly in tertiary hospitals, indicates that higher income may also encourage patients to bypass lower-tier institutions in pursuit of more specialized care. While this reflects improved affordability and access, it may exacerbate the problem of “demand inversion” and pose new challenges to the efficiency of the tiered healthcare delivery structure.

## 6 Robustness

### 6.1 Policy lag effect

According to the *Minimum Wage Regulations*, local minimum wage standards in China have, in principle, been adjusted every 2 years since 2004. It is important to note that the impact of minimum wage adjustments on healthcare expenditures may involve two types of temporal lag. First, there may be institutional delays between the policy announcement and its actual implementation, with variation in timing across regions. Second, even after implementation, the effects on household income, healthcare affordability, and healthcare-seeking behavior may not be immediate, as these responses can take time to materialize. To more accurately capture the policy effects, we use the one-period lagged minimum wage (minimum wage in 2006) in our estimations.^21^

The estimation results using the 2006 minimum wage are presented in Appendix Table A.3. Both OLS and 2SLS estimates show that higher minimum wages significantly increase outpatient, inpatient, and pharmaceutical expenditures at the 1% level, with the strongest effect on inpatient care. The first-stage F-statistics exceed 19.93, the Stock-Yogo critical value for a 10% maximal IV size, indicating strong instrument relevance. The LM statistics reject the null of under-identification across all models, and the Hansen J statistics support the validity of the exclusion restrictions.

### 6.2 Alternative estimation approaches

While IV-2SLS estimators are consistent under homoscedasticity, they may yield biased results in the present of heteroscedasticity. In contrast, the generalized method of moments (GMM) offers greater efficiency (31, 32). Additionally, limited information maximum likelihood (LIML) estimation has been shown to reduce bias arising from weak instrument problems (33). To ensure the robustness of our findings, we re-estimate the model using both two-step GMM and LIML methods. The results are reported in Appendix Table A.4, the estimated coefficients remain qualitatively consistent with the baseline results in both significance and magnitude.

### 6.3 Prefecture-level cities

In China, cities are generally categorized into various administrative levels, including county-level cities, prefecture-level cities, municipalities, etc. The regional policies and healthcare governance systems vary according to these levels. To mitigate potential bias stemming from such urban heterogeneity, we restrict the sample to hospitals located in prefecture-level cities, which account for approximately 70% of all hospitals in the dataset. Panel A of Appendix Table A.5 presents the results. Both OLS and 2SLS estimates remain qualitatively consistent with the baseline findings, suggesting that the main results are not driven by city-level administrative variation.

### 6.4 Hospital maturity

Hospital maturity may also affect the reliability of our results. In the Chinese context, hospitals typically require several years to develop a reputation and attract stable patient flows. Newly established hospitals often face difficulties in building trust and acquiring sufficient demand, which may bias the estimation of healthcare utilization. To address this concern, we exclude hospitals that have been in operation for less than 3 years. Panel B of Appendix Table A.5 reports the results, which remain robust across all specifications. This further supports the validity of our main findings.

## 7 Conclusion

Although the connection between minimum wage policies and healthcare utilization is often noted in policy debates as a potential positive externality, systematic empirical evidence remains scarce. To address this gap, we combine city-level minimum wage data with large national hospital-level microdata from China. This linkage produces a unique dataset that integrates regional policy variation, institutional characteristics, and healthcare-seeking behavior. Using this dataset, we provide the first systematic evidence on how regional minimum wage standards influence residents’ medical expenditures and healthcare utilization, and further reveal their institutional implications for the functioning of China’s hierarchical medical system.

Our findings show that higher minimum wages significantly increase individuals’ medical expenditures. IV estimates indicate that a one-yuan increase in the minimum wage raises outpatient spending per visit by 0.36 yuan, inpatient spending per admission by 7.90 yuan, and pharmaceutical spending per visit by 0.38 yuan. The mechanism analysis points to three primary channels. First, demand for chronic disease treatment: higher minimum wages significantly increase outpatient visits to internal medicine departments. These departments are primarily responsible for managing chronic conditions, suggesting that additional income helps uncover unmet demand for long-term treatment. Second, demand for inpatient treatment and surgery: expenditures on inpatient treatment and surgical services rise significantly and account for the largest increases among all spending categories. Third, preference for higher-quality healthcare: outpatient and pharmaceutical expenditures rise more in for-profit hospitals than in nonprofit ones, indicating a preference for better service environments in routine care. Inpatient expenditures also increase more sharply in tertiary hospitals relative to primary and secondary hospitals, reflecting stronger demand for professional care in higher-tier institutions.

The minimum wage policy has a dual impact on China’s hierarchical medical system. On the one hand, higher minimum wages strengthen the gatekeeping role of primary care facilities by increasing outpatient and pharmaceutical expenditures at primary and secondary hospitals, while also raising outpatient spending in central and western regions where financial barriers have historically been binding. These patterns highlight how income gains can unlock previously unmet demand for common and minor illnesses. On the other hand, higher minimum wages intensify inpatient demand at tertiary hospitals as well as hospitals in the eastern regions where resources are concentrated. This shift reflects patients’ heightened demand for specialized and professional care, but it also contributes to an inverted demand structure, undermining efficiency at higher-tier institutions and aggravating resource misallocation across the system.

This study not only enriches the empirical evidence on the relationship between income policies and healthcare utilization but also broadens our understanding of the structural consequences that income-based interventions can generate for institutional functioning in developing countries. While debates on the broader macroeconomic consequences of minimum wages remain unsettled, our micro-level evidence highlights their positive externalities as a social policy instrument. In particular, given the high level of out-of-pocket payments in China’s healthcare system, income growth directly shapes care-seeking decisions among low-income populations. These results suggest that income policy should receive greater prominence in healthcare reform, especially with regard to its interaction with the hierarchical medical system.

First, minimum wage policies could be integrated with differentiated insurance reimbursement schemes to strengthen the gatekeeping role of primary care facilities. This would involve raising reimbursement rates at community and township hospitals while applying more restrictive reimbursement thresholds at tertiary hospitals, thereby encouraging patients to initiate care at lower levels. Such measures would help reduce the overconcentration of demand at high-level hospitals and improve the efficiency of the hierarchical system. Second, targeted subsidies should be provided for low-income groups, particularly for long-term expenditures such as chronic disease management and inpatient care. These subsidies should be coordinated with minimum wage adjustments to reduce financial burdens and enhance both access to and reliance on basic healthcare services. Finally, insurance reforms should account for regional heterogeneity. In eastern regions, priority should be given to optimizing referral and diversion mechanisms, while in central and western regions, efforts should focus on strengthening the capacity of primary care facilities. Dynamic adjustment of insurance parameters across regions would further improve resource allocation and enhance the resilience of the hierarchical medical system.

Beyond its relevance for China, this study offers broader implications for global health governance and institutional design. Many LMICs operate under healthcare systems characterized by structural constraints such as fragmented service delivery, unequal access, and persistent underutilization of primary care. Our findings show that income-based policy shocks—even when originating outside the health sector—can exacerbate these systemic weaknesses. In particular, the concentration of demand in higher-tier and for-profit hospitals suggests that income gains may reinforce rather than mitigate institutional inefficiencies. Furthermore, the inertia in patients’ healthcare-seeking behavior points to strong path dependence in system responses, underscoring the limited adaptability of health institutions to exogenous shocks. These insights highlight the importance of integrated, cross-sectoral strategies in health policymaking, especially in settings where health systems remain vulnerable to income and labor market dynamics.

For future research, further work could examine the effects of minimum wage increases on the health outcomes of other household members and explore heterogeneous across income groups—for example, how wage changes affect the healthcare behaviors of children, parents, and high-income earners. Additional studies might assess the impact of minimum wage policies on chronic disease recovery and overall labor productivity. From a broader perspective, future research could also investigate how developing countries can improve the efficiency of healthcare systems and ensure equitable access to medical services in the context of income redistribution and poverty alleviation policies.

## Data Availability

The raw data supporting the conclusions of this article will be made available by the authors, without undue reservation.

## Footnotes

^1^According to the statistics from Ministry of Health of the People’s Republic of China (MOH), by the end of 2025, the coverage rate of basic medical insurance in China is expected to remain above 95%.

^2^The New Healthcare Reform, launched in 2009, is a comprehensive policy initiative aimed at improving the accessibility, quality, and equity of healthcare services in China. It focuses on strengthening primary healthcare infrastructure, expanding insurance coverage, and reducing costs for medicines and medical services to enhance the national health system.

^3^Primary hospitals comprise community-level first-tier hospitals, health centers, community health service centers, and township hospitals.

^4^In the national hospital dataset used in this study, there are 13,274 primary hospitals, accounting for 61% of the total hospital sample.

^5^China has lifted 750 million people out of poverty through the implementation of targeted poverty alleviation strategies, contributing more than 70% to global poverty reduction efforts. By 2020, it had completely eradicated absolute poverty, achieving the UN’s 2030 Sustainable Development Goal on poverty reduction a decade ahead of schedule.

^6^The data does not include sanatoriums, specialized disease prevention and treatment hospitals, or small clinics.

^7^Non-profit hospitals and most for-profit hospitals above a certain scale are required to be rated in China. Unrated hospitals are typically small typically or for-profit clinics, due to their failure to meet the established rating standards. Specifically, within the sample analyzed in this study, hospitals with a rating have an average of 275 beds, while unrated hospitals average only 70 beds.

^8^Following the exclusion of observations with missing control variables, 240 city-level samples are retained for estimation.

^9^According to official documents from provincial and municipal governments, all cities in our sample had launched the Urban Resident Basic Medical Insurance pilot program by 2008. As a result, the implementation status does not vary across observations and is not included as a separate covariate in the regressions.

^10^The share of older adults refers to the proportion of the population aged 65 and above.

^11^The education rate is defined as the proportion of residents aged 15 and above who have received formal education.

^12^Ethnic minority region is a dummy variable equal to 1 if a city is either an autonomous prefecture or administratively part of an autonomous region, and 0 otherwise.

^13^Data on regional characteristics are sourced from the *China Urban Statistical Yearbook* and official local government sources.

^14^For the method of variable calculation, we refer to an analysis Report of National Health Services Survey in China issued by the MOH.

^15^To balance sample size and estimation consistency, we adopt an available-case approach when handling missing values in outcome and mechanism variables. This method retains all valid observations for each variable, rather than applying listwise deletion across all variables. This approach improves data utilization in large-scale datasets with partial missingness, though the number of observations may vary across variables.

^16^Although the Ministry of Labor and Social Security in China issued the *Regulation on Minimum Wages for Enterprises* in 1993 as an administrative rule, the minimum wage system was not enforceable for local governments or enterprises prior to 2004, raising doubts about the accuracy and implementation of the actual minimum wage data.

^17^Because the city itself cannot be excluded from the province-level average in municipalities directly under the central government (including Beijing, Shanghai, Tianjin, and Chongqing), hospitals in these cities are excluded from the construction of *IV_2_*. In addition, due to substantial missing wage data in ethnic minority regions such as Xinjiang and Tibet, hospitals from these two regions are also excluded from the calculation of *IV_2_* to ensure the robustness of our estimation.

^18^Data are from the 2008 Statistical Bulletin on the Development of China’s Health Sector, issued by the MOH.

^19^Appendix Tables A.1, A.2 report the corresponding OLS estimates for inpatient and outpatient categories, and the results remain robust.

^20^In the nationwide hospital data used in this paper, for-profit hospitals account for only 18.9% of the total number of hospitals in China.

^21^For simplicity, potential anticipatory effects are not considered.
