# Supplementary material for: The impact of minimum wages on medical expenditures and resource misallocation: evidence from China’s healthcare system
Source: Front Public Health. 2025 Nov 4;13:1646631. doi: 10.3389/fpubh.2025.1646631 (PMC12623400; doi:10.3389/fpubh.2025.1646631)
Supplement: Supplementary file 1 [file Table_1.docx]

**Appendix**

**Table A.1**

Effects on inpatient expenditure per admission by category: OLS estimation.

| Variables | (1) | (2) | (3) | (4) | (5) | (6) |
| --- | --- | --- | --- | --- | --- | --- |
|  | Beds expenditure | Examination expenditure | Inspection expenditure | Laboratory test expenditure | Treatment expenditure | Surgery expenditure |
| Minimum wage | 1.2576*** | 0.2397*** | 0.4918*** | 0.4923*** | 2.1171*** | 2.2487*** |
|  | (0.1155) | (0.0363) | (0.0373) | (0.0333) | (0.1506) | (0.2829) |
| Observations | 14,035 | 11,917 | 13,870 | 13,587 | 14,209 | 11,006 |
| Province FE | Yes | Yes | Yes | Yes | Yes | Yes |
| Hospital Controls | Yes | Yes | Yes | Yes | Yes | Yes |
| City controls | Yes | Yes | Yes | Yes | Yes | Yes |

*Notes:* Only IV estimation results are presented. ***p < 0.01, **p < 0.05, *p < 0.1. The dependent variable measures outpatient expenditure per visit by category. Numbers in parentheses are robust standard errors. Hospitals located in municipalities directly under the central government and Tibet Autonomous Region are excluded from the IV regressions. The Stock-Yogo weak identification test critical value for 10% maximal IV size is 19.93.

**Table A.2**

Effects on outpatient expenditure per visit by category: OLS estimation.

| Variables | (1) | (2) | (3) | (4) | (5) | (6) |
| --- | --- | --- | --- | --- | --- | --- |
|  | Registration expenditure | Examination expenditure | Inspection expenditure | Laboratory test expenditure | Treatment expenditure | Surgery expenditure |
| Minimum wage | 0.0026*** | 0.0087*** | 0.0073 | 0.0205*** | 0.1092*** | 0.0506*** |
|  | (0.0008) | (0.0025) | (0.0054) | (0.0043) | (0.0111) | (0.0097) |
| Observations | 13,465 | 12,438 | 14,930 | 14,488 | 15,098 | 12,872 |
| Province FE | Yes | Yes | Yes | Yes | Yes | Yes |
| Hospital Controls | Yes | Yes | Yes | Yes | Yes | Yes |
| City controls | Yes | Yes | Yes | Yes | Yes | Yes |

*Notes:* Only IV estimation results are presented. ***p < 0.01, **p < 0.05, *p < 0.1. The dependent variable measures outpatient expenditure per visit by category. Numbers in parentheses are robust standard errors. Hospitals located in municipalities directly under the central government and Tibet Autonomous Region are excluded from the IV regressions. The Stock-Yogo weak identification test critical value for 10% maximal IV size is 19.93.

**Table A.3**

Robustness to policy lag effect.

| Variables | (1) | (2) | (3) | (4) | (5) | (6) | |
| --- | --- | --- | --- | --- | --- | --- | --- |
|  | OLS | IV | OLS | IV | OLS | IV | |
|  | Outpatient expenditure per visit | | Inpatient expenditure per admission | | Pharmaceutical expenditure per visit | |  |
| Minimum wage in 2006 | 0.3378*** | 0.4138*** | 7.4622*** | 9.1691*** | 0.3455*** | 0.4361*** | |
|  | (0.0511) | (0.0693) | (0.4699) | (0.5756) | (0.0474) | (0.0562) | |
| Observations | 15,950 | 14,505 | 14,946 | 13,661 | 15,897 | 14,452 | |
| Hospital-level controls | Yes | Yes | Yes | Yes | Yes | Yes | |
| City-level controls | Yes | Yes | Yes | Yes | Yes | Yes | |
| Province FE | Yes | Yes | Yes | Yes | Yes | Yes | |
| First stage F-stats |  | 13607.72 |  | 13028.55 |  | 13559.215 | |
| p value of LM statistic |  | 0.0000 |  | 0.0000 |  | 0.0000 | |
| p value of Hansen J statistic |  | 0.2584 |  | 0.3434 |  | 0.8873 | |

*Notes:* ***p < 0.01, **p < 0.05, *p < 0.1. Numbers in parentheses are robust standard errors. Hospitals located in municipalities directly under the central government and Tibet Autonomous Region are excluded from the IV regressions. The Stock-Yogo weak identification test critical value for 10% maximal IV size is 19.93.

**Table A.4**

Robustness to alternative estimation approaches.

| Variables | (1) | (2) | (3) | (4) | (5) | (6) |
| --- | --- | --- | --- | --- | --- | --- |
|  | GMM | LIML | GMM | LIML | GMM | LIML |
|  | Outpatient expenditure per visit | | Inpatient expenditure per admission | | Pharmaceutical expenditure per visit | |
| Minimum wage | 0.3505*** | 0.3553*** | 7.9141*** | 7.8954*** | 0.3750*** | 0.3752*** |
|  | (0.0594) | (0.0596) | (0.4940) | (0.4946) | (0.0483) | (0.0484) |
| Observations | 14,505 | 14,505 | 13,661 | 13,661 | 14,452 | 14,452 |
| Hospital-level controls | Yes | Yes | Yes | Yes | Yes | Yes |
| City-level controls | Yes | Yes | Yes | Yes | Yes | Yes |
| Province FE | Yes | Yes | Yes | Yes | Yes | Yes |
| First stage F-stats | 10454.479 | 10454.479 | 9784.3537 | 9784.3537 | 10406.183 | 10406.183 |
| p value of LM statistic | 0.0000 | 0.0000 | 0.0000 | 0.0000 | 0.0000 | 0.0000 |
| p value of Hansen J statistic | 0.232 | 0.232 | 0.4559 | 0.4559 | 0.8198 | 0.8198 |

*Notes:* ***p < 0.01, **p < 0.05, *p < 0.1. Numbers in parentheses are robust standard errors. Hospitals located in municipalities directly under the central government and Tibet Autonomous Region are excluded from the IV regressions. The Stock-Yogo weak identification test critical value for 10% maximal IV size is 19.93.

**Table A.5**

Robustness to sample selection.

| Variables | (1) | (2) | (3) | (4) | (5) | (6) |
| --- | --- | --- | --- | --- | --- | --- |
|  | OLS | IV | OLS | IV | OLS | IV |
|  | Outpatient expenditure per visit | | Inpatient expenditure per admission | | Pharmaceutical expenditure per visit | |
| **Panel A: Prefecture-level city** | |  |  |  |  |  |
| Minimum wage | 0.1816*** | 0.2414*** | 4.9326*** | 6.5583*** | 0.2080*** | 0.3098*** |
|  | (0.0388) | (0.0490) | (0.3782) | (0.5420) | (0.0357) | (0.0471) |
| Observations | 11,139 | 11,139 | 10,633 | 10,633 | 11,110 | 11,110 |
| Hospital-level controls | Yes | Yes | Yes | Yes | Yes | Yes |
| City-level controls | Yes | Yes | Yes | Yes | Yes | Yes |
| Province FE | Yes | Yes | Yes | Yes | Yes | Yes |
| First stage F-stats |  | 10101.1 |  | 9672.9109 |  | 10074.421 |
| p value of LM statistic |  | 0.0000 |  | 0.0000 |  | 0.0000 |
| p value of Hansen J statistic |  | 0.127 |  | 0.9431 |  | 0.2475 |
| **Panel B: Hospital age>3** | |  |  |  |  |  |
| Minimum wage | 0.2361*** | 0.3230*** | 6.0365*** | 7.7996*** | 0.2582*** | 0.3515*** |
|  | (0.0413) | (0.0619) | (0.4050) | (0.5221) | (0.0362) | (0.0433) |
| Observations | 13,981 | 12,748 | 13,207 | 12,095 | 13,937 | 12,706 |
| Hospital-level controls | Yes | Yes | Yes | Yes | Yes | Yes |
| City-level controls | Yes | Yes | Yes | Yes | Yes | Yes |
| Province FE | Yes | Yes | Yes | Yes | Yes | Yes |
| First stage F-stats |  | 9252.1715 |  | 8678.2536 |  | 9211.9471 |
| p value of LM statistic |  | 0.0000 |  | 0.0000 |  | 0.0000 |
| p value of Hansen J statistic |  | 0.2597 |  | 0.5617 |  | 0.6628 |

*Notes:* ***p < 0.01, **p < 0.05, *p < 0.1. Numbers in parentheses are robust standard errors. Hospitals located in municipalities directly under the central government and Tibet Autonomous Region are excluded from the IV regressions. The Stock-Yogo weak identification test critical value for 10% maximal IV size is 19.93.
